# A case report of delayed-diagnosed adult multisystem Langerhans cell histiocytosis with isolated MAP2K1 somatic mutation and systematic literature review

**DOI:** 10.3389/fonc.2026.1885471

**Published:** 2026-09-07

**Authors:** Qijian Zhang, Ping He, Yang Dong, Lixiang Yang, Junfei Shao, Weiyi Huang

**Affiliations:** The Affiliated Wuxi People’s Hospital of Nanjing Medical University, Wuxi, China

**Keywords:** adult clinical case, Langerhans cell histiocytosis, MAP2K1 mutation, multisystem involvement, targeted therapy

## Abstract

Numerous case reports regarding adult multisystem Langerhans cell histiocytosis (MS-LCH) have been published, yet the vast majority only describe single-organ involvement. Cases presenting sequential multi-organ lesions, clinical manifestations mimicking idiopathic hypophysitis and refractory gastritis, accompanied by long-term missed diagnosis and delayed definitive confirmation remain extremely rare. This article reports a 44-year-old female patient with delayed-diagnosed multisystem LCH. Her initial symptom was isolated central diabetes insipidus, which was misdiagnosed as infundibulohypophysitis. Subsequently, she developed intractable gastrointestinal discomfort accompanied by unexplained 10 kg weight loss. The definitive diagnosis was established nearly 10 months after symptom onset until a subcutaneous frontal skull mass emerged. Pathological diagnosis was obtained via surgical resection. The patient received cytarabine induction chemotherapy followed by salvage therapy combining TCD regimen and denosumab, achieving sustained partial remission of systemic lesions. Targeted next-generation sequencing identified a grade II somatic MAP2K1 mutation without the common BRAF V600E hotspot mutation, providing definitive molecular evidence for long-term maintenance targeted therapy with MEK inhibitors. This case is intended to deepen clinicians’ recognition on the diagnostic and therapeutic strategies of adult LCH, facilitate early confirmed diagnosis and timely clinical intervention to reduce related complications, and further explore its underlying pathogenesis as well as individualized management regimens.

## 1 Introduction

Langerhans cell histiocytosis is a rare clonal histiocytic disorder that predominantly affects pediatric populations, with an estimated adult incidence of 1 to 2 cases per million population annually⁠ (1). Its clinical manifestations present prominent heterogeneity, ranging from localized solitary bone lesions to life-threatening multiorgan damage, which greatly hinders early clinical identification and diagnosis (2). With the rapid progress of molecular pathological research in recent years, novel perspectives have been provided for the diagnostic evaluation and standardized treatment of LCH⁠ (3). Most published case reports of adult LCH only document solitary bone lesions or isolated pituitary lesions. Cases with sequential combined involvement of endocrine, digestive, cranial, mediastinal and multiple systemic skeletal organs accompanied by prolonged missed diagnosis are scarce. The atypical disease progression of this patient is highly likely to be misdiagnosed as common endocrine and digestive diseases, delivering unique clinical teaching value for frontline physicians across multiple departments. In addition, MAPK pathway aberrations represent the core driver of LCH, and MAP2K1-mutant LCH requires differentiated targeted management distinct from BRAF V600E-mutant subtypes (15). On this basis, this study reported one adult case of multisystem LCH, and further discussed the diagnostic dilemmas and therapeutic challenges by reviewing relevant published literature.

## 2 Case description

### 2.1 Chief complaint and medical history

A 44-year-old female patient was admitted to our department on May 14, 2024. She complained of persistent thirst with polydipsia and polyuria for one year, as well as a newly detected mass in the right frontal region within the latest two weeks.

As early as August 2023, she visited the Endocrinology Department of our hospital owing to three months of unrelenting thirst, polydipsia and polyuria. During hospitalization, water deprivation test confirmed complete central diabetes insipidus. Pituitary magnetic resonance imaging (MRI) scanned on September 2, 2023 displayed abnormal signal changes in the pituitary stalk and posterior pituitary lobe, suggesting potential pituitary hypophysitis. Auxiliary examinations including echocardiography, lower extremity Doppler ultrasound, renal-ureteral-bladder ultrasonography and cervical lymph node ultrasound revealed no remarkable abnormal findings. The endocrinology team prescribed desmopressin at a dose of 0.05 mg per 8 hours for symptomatic control. After intervention, her daily urine output was controlled within 3–4 L with only 0–1 nocturia episodes, and she was discharged with stable condition.

On September 15, 2023, the patient attended Huashan Hospital Affiliated to Fudan University for further consultation. Repeated pituitary MRI showed a full pituitary gland with a height of about 7 mm and relatively homogeneous internal signals, without obvious focal abnormal lesions. The pituitary stalk was thickened with a maximum diameter of roughly 4 mm. No obvious downward displacement of the sella floor was observed, and no abnormal manifestations were found in the suprasellar and parasellar regions. Contrast-enhanced scanning showed no significant abnormal enhancement. Clinically, hypothalamic-infundibular neurohypophysitis was highly suspected. Laboratory indicators including IgG4, ACE, T-SPOT, AFP and hCG were all within normal reference ranges. Systemic screening covering chest CT, whole-body skeletal plain radiography, superficial lymph node ultrasound and contrast-enhanced liver MRI showed no positive lesions. Desmopressin was adjusted to 0.1 mg three times a day to maintain urinary control.

Starting in late September 2023, the patient gradually developed gastric discomfort accompanied by recurrent nausea and vomiting. On November 11, 2023, she received symptomatic treatment with itopride hydrochloride and compound azintamide enteric-coated tablets in Ruijin Hospital, whereas her symptoms obtained no obvious relief. On March 18, 2024, she presented to the First Affiliated Hospital of Soochow University. Gastroscopy indicated superficial gastritis, and subsequent symptomatic medication with compound azintamide enteric-coated tablets combined with bifidobacterium preparation still failed to alleviate her gastrointestinal symptoms. Throughout this period, a definitive etiology could not be identified, and the patient’s body weight dropped by around 10 kg.

In early May 2024, the patient accidentally found a painless subcutaneous mass in the right frontal region without obvious inducement. The lesion was small and non-erythematous at the beginning, yet it gradually enlarged and became tender thereafter. Head CT examination in our hospital demonstrated slight hyperdense nodular lesion in the pituitary stalk area, thickened soft tissue with heterogeneous density in the right frontal scalp, and hypodense focal bone destruction in the adjacent frontal bone. She was then hospitalized for further etiological exploration.

**Figure 1:**
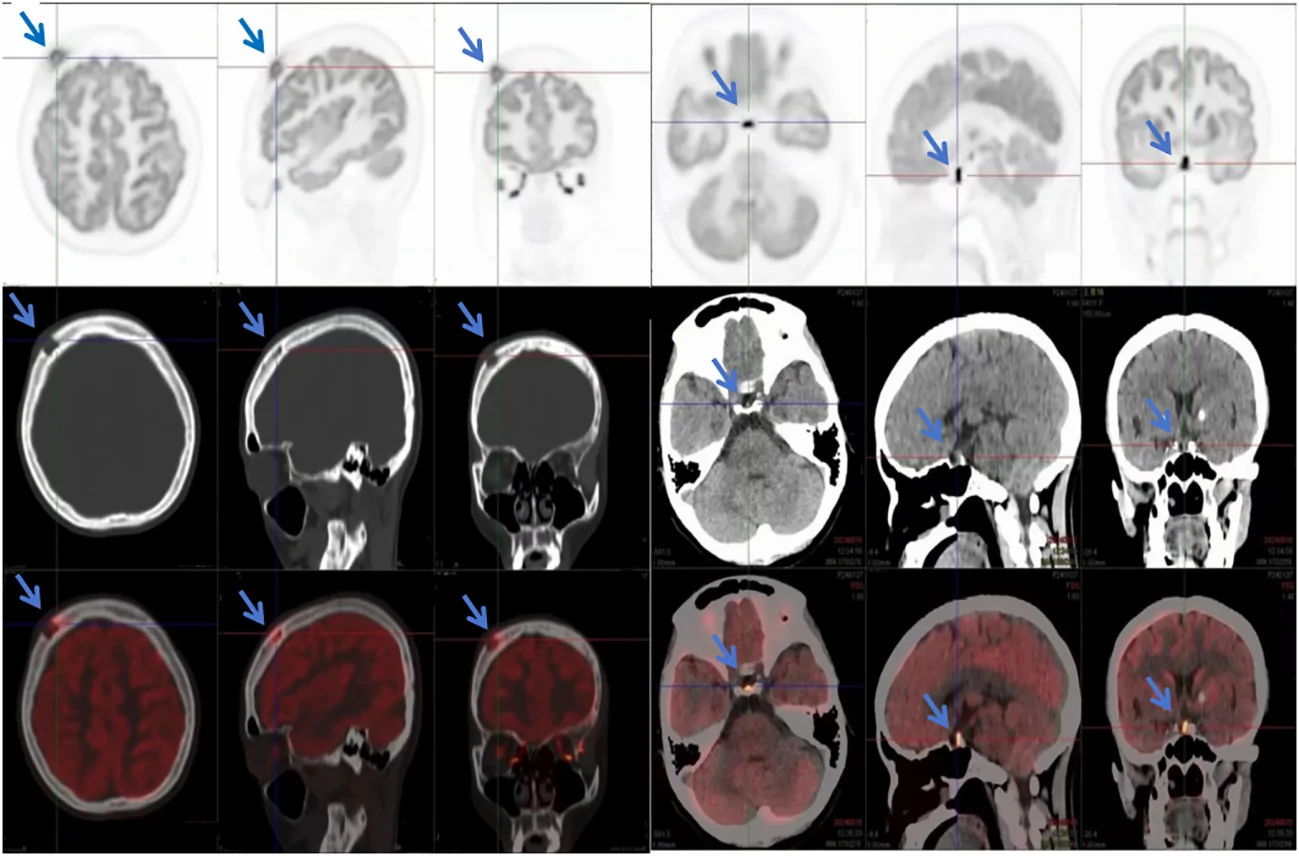
Gray scale = CT brain; Red overlay = FDG-PET hypermetabolism. The areas marked by blue arrows represent hypermetabolic lesions. The left side shows the skull lesion, and the right side displays the pituitary lesion.

**Figure 2:**
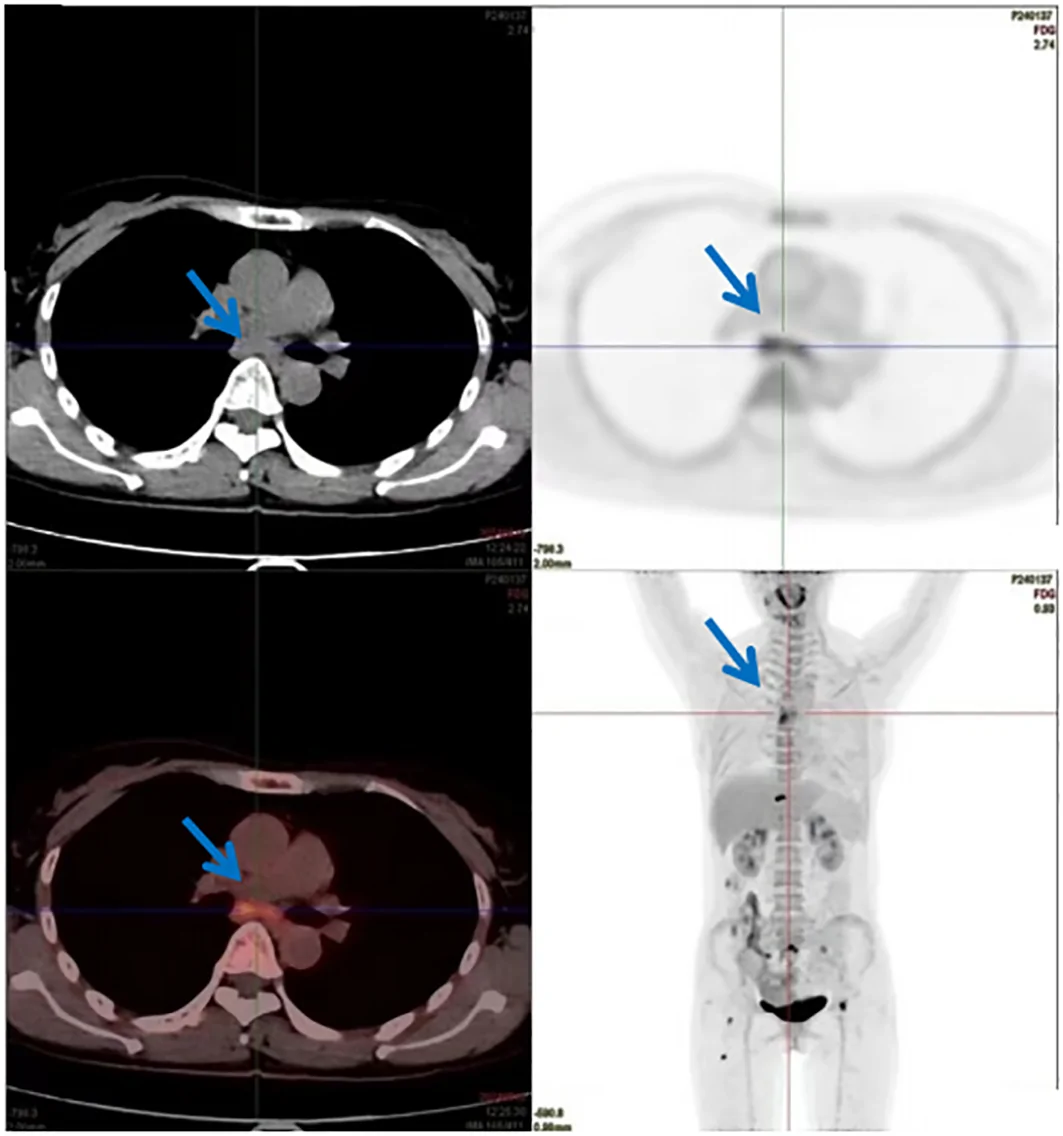
Axial CT, PET, fused PET/CT and whole-body PET maximum intensity projection (MIP) images. The blue arrow points to the enlarged mediastinal lymph node with high metabolic activity.

PET-CT examination yielded the following key findings:

1. Focal bone defect in the right frontal region with abnormally elevated glucose metabolism; increased metabolic activity in the pituitary stalk and posterior pituitary lobe (**Figure 1**), highly suggestive of eosinophilic granuloma.
2. Posterior mediastinal space-occupying lesion with mildly increased glucose metabolism (**Figure 2**); solid nodule detected in the upper lobe of the left lung; locally elevated metabolic foci observed in the 12th thoracic vertebra, sacrum, left acetabulum, bilateral femoral necks and proximal right femur. Metastatic malignant tumor originating from the posterior mediastinum could not be completely excluded.

**Figure 3:**
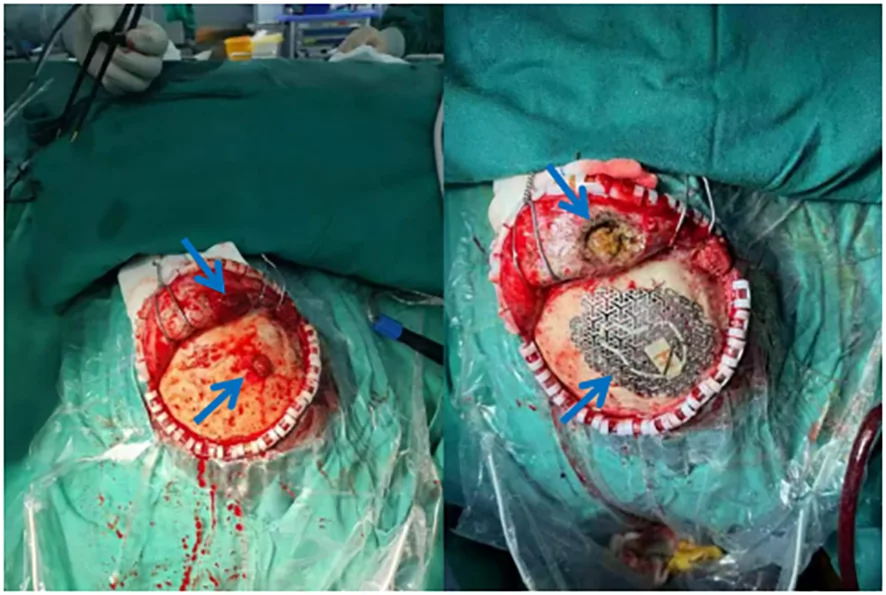
Intraoperative photographs. Left: after left craniotomy, the lesion was exposed; the blue arrows mark the areas where the tumor invaded the scalp and cranial bone. Right: titanium mesh cranioplasty was completed; the blue arrows indicate the resected scalp tumor and the titanium mesh implanted to repair the cranial bone defect after tumor resection.

Considering the high biopsy risk of the mediastinal lesion, we performed surgical resection and biopsy for the skull lesion. Intraoperative exploration showed that the tumor tissue had broken through the outer table of the skull and invaded local scalp soft tissues. The affected skull and involved scalp tissues were completely excised, and skull defect reconstruction was conducted with titanium mesh implantation (**Figure 3**).

**Figure 4:**
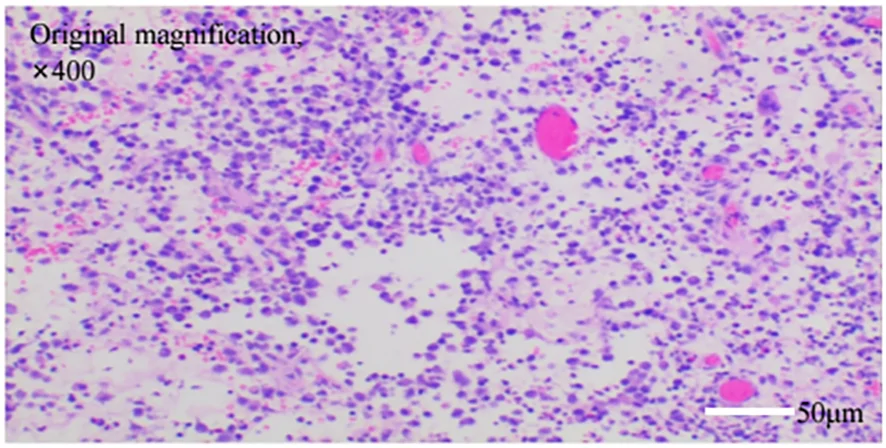
Hematoxylin and eosin (HE) staining of Langerhans cell histiocytosis lesion. Proliferating Langerhans cells with characteristic nuclear grooves were diffusely infiltrated with abundant eosinophils, lymphocytes and multinucleated giant cells. Original magnification, ×400; scale bar = 50 μm.

Postoperative histopathological staining showed massive infiltration of lymphocytes and eosinophils, accompanied by proliferation of histiocyte-like cells and multinucleated giant cells with characteristic nuclear grooves (**Figure 4**). Immunohistochemical examination (Case No. I24-02343) presented: CD1a (+), S-100 (+), Ki-67 (approximately 25% positive), CD30 (-), CD68 (partial positivity), SATB2 (-), AE1/AE3 (-), CD99 (-). Combined with morphological and immunohistochemical features, the lesion was confirmed as Langerhans cell histiocytosis (4).

After pathological confirmation, the patient consulted the Hematology Department of Peking Union Medical College Hospital on June 27, 2024. Chemotherapy was initiated with ARA-C regimen: cytarabine 0.13 g combined with 2 mL 0.9% normal saline via subcutaneous injection once daily for 5 consecutive days, with a treatment cycle of 35 days. Oral ondansetron hydrochloride 4 mg per day was given synchronously for antiemetic support.Next-generation sequencing molecular testing completed on July 12, 2024 showed negative BRAF V600E hotspot mutation, with only a somatic MAP2K1 variant identified: NM_002755.4:exon3:c.299_309delinsTCTGGAGA:p.H100_I103delinsLWR. This variant was classified as Tier II according to the AMP/ASCO/CAP guidelines for somatic variant interpretation (5).

After six cycles of chemotherapy, the patient’s nausea and vomiting were remarkably relieved, and her diabetes insipidus symptoms were well controlled. Re-examined PET-CT in an external hospital revealed no abnormal hypermetabolic foci or residual soft tissue mass in the right frontal operative area, pituitary stalk and posterior pituitary lobe. Multiple skeletal lesions showed obvious improvement compared with previous imaging, while the subcarinal node in the mediastinum remained stable.Therapeutic response was evaluated based on the PET-CT response criteria (PRC) from the 2025 Chinese Expert Consensus on Adult Langerhans Cell Histiocytosis and standardized adult LCH efficacy grading standards modified from pediatric Histiocyte Society criteria and validated in Chinese adult patient cohorts (12). Lesion response stratification was as follows: complete response (CR) for pituitary and frontal bone lesions, partial response (PR) for systemic multifocal skeletal lesions, and stable disease (SD) for subcarinal mediastinal lymph nodes with persistent residual metabolically active lesions. The persistent mediastinal lesion constituted the core indication for switching to salvage TCD combination therapy. In addition, denosumab was added to inhibit osteolytic damage due to concurrent bone destruction.

Accordingly, on January 26, 2025, the patient received further treatment at the Cancer Hospital, Chinese Academy of Medical Sciences. The therapeutic regimen was adjusted to TCD protocol combined with denosumab: thalidomide 100 mg daily, cyclophosphamide at a dose of 400 mg, and dexamethasone 40 mg administered on the 1st, 8th, 15th and 22nd day, with a repeated cycle of 4 weeks.

After another six-month standardized chemotherapy course, her diabetes insipidus was effectively controlled, gastric discomfort was significantly alleviated, and body weight recovered obviously. Follow-up PET-CT rechecked on July 21, 2025 showed no evidence of tumor recurrence in the right frontal operative region, and the metabolic activity of mediastinal subcarinal lymph node was decreased compared with the last examination.

## 3 Discussion

### 3.1 Diagnostic difficulties of adult LCH

Adult LCH often involves multiple organ systems, among which bone tissue, pituitary gland and lymph nodes are the most commonly affected sites (6). It is frequently misdiagnosed as single-system diseases such as primary bone tumor or isolated hypophysitis. Skull bone represents the most predilection site of skeletal involvement, while pituitary lesion is the dominant central nervous system manifestation, usually presenting as central diabetes insipidus⁠ (7). This patient experienced nearly 10 months of diagnostic delay attributable to four core factors:

1. Atypical isolated initial endocrine manifestation: Exclusive presentation of central diabetes insipidus without osteolytic destruction or lymphadenopathy, closely mimicking idiopathic infundibulohypophysitis.
2. Non-specific secondary gastrointestinal symptoms: Recurrent nausea and vomiting with only mild superficial gastritis on gastroscopy, lacking pathognomonic diagnostic clues for LCH.
3. Limitations of early routine screening modalities: Conventional CT, skeletal radiography and ultrasound failed to detect occult mediastinal and multifocal skeletal lesions prior to emergence of the cranial mass.
4. Insufficient vigilance across clinical disciplines: Endocrinologists and gastroenterologists lacked adequate awareness of this rare clonal histiocytic proliferative disorder, limiting interventions to symptomatic management without systematic rare disease etiological screening.

The typical pathological feature of LCH is the presence of characteristic Langerhans cells with nuclear grooves and abundant eosinophilic cytoplasm, usually mixed with infiltrated eosinophils, lymphocytes and multinucleated giant cells. Immunohistochemically, positive expression of CD1a, CD207 (Langerin) and S-100 serves as the core diagnostic biomarker of LCH. Pathological confirmation is of decisive significance for definitive diagnosis and subsequent formulation of treatment plans (4).

### 3.2 Research progress on molecular mechanisms

Advancements in genetic studies have demonstrated that BRAF V600E mutation can be detected in approximately 50%–60% of LCH cases, and somatic MAP2K gene mutations account for about 25% of all patients. Moreover, rare genetic alterations including ARAF, ERBB3, NRAS and KRAS mutations have also been reported in partial adult LCH lesions⁠ (5, 10). Consistent with large adult LCH cohort molecular profiling data, this patient harbored no BRAF V600E mutation and carried only a Tier II MAP2K1 variant. This MAP2K1 mutation constitutively activates MEK signaling independent of upstream BRAF regulation, driving clonal proliferation of Langerhans cells. This mutational subtype accounts for one-quarter of adult LCH cases and represents the primary candidate population for MEK inhibitor targeted therapy. The detection of MAP2K1 mutation in this patient provides reliable molecular evidence for subsequent targeted therapy selection (11). In general, molecular genetic detection greatly promotes the precision diagnosis and individualized treatment of LCH by identifying abnormal mutations within the MAPK signaling pathway.

### 3.3 Updated understanding of therapeutic strategies

The traditional VP regimen consisting of vinca alkaloids combined with prednisone is the first-line standard scheme for pediatric LCH, whereas it shows limited clinical efficacy in adult populations (6).

For adult patients with newly diagnosed multisystem or multifocal single-system LCH, cytarabine monotherapy or cytarabine-based combined regimens are currently recommended as preferred initial treatment.

For patients with solitary bone lesion, regular follow-up assessment every three months is acceptable as initial management. Further intervention strategies including continuous observation, local lesion treatment or systematic chemotherapy are determined according to re-evaluation results. Local therapeutic options involve lesion curettage, surgical excision and local radiotherapy. Denosumab application can be considered for patients with solitary bone lesion combined with other organ involvement or multifocal bone lesions with mild clinical symptoms (10, 12).

For relapsed and refractory adult LCH cases, the TCD regimen (thalidomide plus cyclophosphamide 300 mg/m² plus dexamethasone) is suggested for 12 treatment cycles, followed by 12-month maintenance therapy with single-agent thalidomide⁠ (12). In addition, clofarabine monotherapy has also achieved certain clinical benefits in partial refractory patients.

Furthermore, targeted intervention with BRAF inhibitors is applicable to BRAF V600E mutation-positive LCH patients, while MEK inhibitors can be adopted for those with MAP2K mutation and negative BRAF V600E status (11, 15).

### 3.4 Clinical significance of MAP2K1 mutation and corresponding targeted therapy

1. Epidemiological profile: Approximately 25% of adult LCH patients carry MAP2K1 mutations, almost exclusively within the BRAF V600E-negative subgroup;
2. Pathogenic mechanism: MAP2K1 variants induce constitutive MEK kinase activation, driving Langerhans cell clonal proliferation via a BRAF-independent signaling cascade;
3. Targeted therapeutic options: Domestic and international expert consensus recommends MEK inhibitors (trametinib, cobimetinib) for relapsed/refractory MAP2K1-mutant adult LCH (11); published case reports have verified durable lesion regression after trametinib administration in MAP2K1 deletion-variant LCH patients (15).
4. Long-term management strategy for this patient: Maintenance targeted therapy with MEK inhibitors is planned upon completion of standard TCD chemotherapy to mitigate long-term relapse risk.
5. Existing limitations: Clinical data supporting MEK inhibitors for adult multisystem LCH are limited to small case series; large-scale cohort follow-up studies are required to characterize long-term safety, adverse event monitoring and sustained therapeutic efficacy. Despite existing potential adverse reactions and unclear long-term curative effect, targeted therapy provides a promising survival opportunity for refractory and recurrent LCH patients⁠ (11).

### 3.5 Prognostic influencing factors

The 5-year overall survival rate of adult multisystem LCH is about 75%. High-risk prognostic factors include liver or lung involvement and poor early response to treatment⁠ (13). Replacement therapy for pituitary lesion-related endocrine disorder is crucial for improving patients’ life quality (7).

The prognosis of LCH is comprehensively determined by lesion scope, high-risk organ involvement, gene mutation status and treatment response. The 5-year survival rate of unifocal LCH exceeds 90% (14). Multisystem LCH accompanied with high-risk organ involvement such as liver, spleen and bone marrow usually presents a poor prognosis, requiring timely systematic chemotherapy or targeted intervention. A landmark systematic review confirmed that multi-organ involvement and recurrent disease are independent predictors of inferior overall survival in adult LCH (17). Clinical outcomes vary greatly depending on treatment response and involved organs in adult patients, ranging from complete recovery without sequelae to severe irreversible organ dysfunction. This case fully proves that early diagnosis and molecular screening of gene mutations exert important value in optimizing the prognosis of multisystem LCH patients.

### 3.6 Clinical implications for multidisciplinary frontline physicians

1. Endocrinologists: In adult patients with unexplained central diabetes insipidus and pituitary stalk thickening suboptimally controlled by desmopressin replacement, early whole-body PET-CT is indicated to rule out occult multisystem LCH (8).
2. Gastroenterologists: Patients with refractory chronic gastrointestinal symptoms, unexplained weight loss and concurrent pituitary abnormalities require comprehensive rare disease screening rather than isolated symptomatic gastrointestinal management.
3. Surgeons and radiologists: For patients with chronic pituitary lesions complicated by new-onset painless subcutaneous cranial masses, surgical biopsy with complete pathological and immunohistochemical testing should be prioritized for definitive diagnosis (9).
4. Hematologists: All adult multisystem LCH patients should undergo MAPK pathway next-generation sequencing if sequencing platforms are available, to guide individualized salvage chemotherapy and targeted therapy.
5. Therapeutic reference: Cytarabine induction followed by TCD combined with denosumab constitutes an effective salvage regimen for adult multisystem LCH complicated by multifocal osteolytic lesions and residual mediastinal disease.

## 4 Conclusion

Adult LCH presents heterogeneous clinical manifestations and tends to develop systemic multiorgan involvement. Early definite diagnosis relies on the combination of pathological examination and molecular genetic detection. Systemic whole-body screening is indispensable even for patients initially diagnosed with solitary lesion at early stage. PET-CT plays an irreplaceable role in identifying hidden systemic metastatic lesions, and genetic detection has also become a necessary examination item (8). Targeted therapy targeting BRAF mutation yields favorable prognostic benefits, while further clinical exploration is still needed regarding its long-term efficacy and drug resistance problem (16). For MAP2K1-mutant, BRAF V600E-negative adult multisystem LCH, long-term maintenance targeted therapy with MEK inhibitors represents a promising individualized management strategy, warranting further validation through large-scale prospective clinical trials (15).
